# A miniaturization event in Ornithodira

**DOI:** 10.1038/s42003-020-01236-1

**Published:** 2020-09-07

**Authors:** Luke R. Grinham

**Affiliations:** Communications Biology, https://nature.com/commsbio

## Abstract

The origins of Ornithodira (the last common ancestor of dinosaurs, pterosaurs, and their descendants) are yet to be resolved, and have the potential to inform on the diversification of some of the most intriguing ecologies and body forms to evolve in reptiles. A recent discovery of an extremely small ornithodiran archosaur by Christian Kammerer and colleagues is indicative of a miniaturisation event early in the evolution of Ornithodira. This raises questions about the evolution of characters associated with small body forms in these groups, such as flight and body surface integument.

Ornithodira has a complex and colourful history in palaeontology. With gargantuan sauropods, independent evolutions of powered flight in pterosaurs and birds, and all manner of ecological niches filled, the total clade exudes diversity. Evolutionary relationships and biological interpretations are frequently debated and reassessed.

Work from Kammerer et al.^1^ provides the most recent contribution to the debate surrounding the origins of Ornithodira by describing a new species of lagerpetid, *Kongonaphon kely*. With an estimated height of just 10 cm, this new specimen represents one of the smallest non-avian ornithodirans ever described, and is confidently diagnosed by the authors as a non-perinate. This tiny body is likely representative of a grown individual. Following a phylogenetic analysis conducted in PAUP*, the authors performed an ancestral state reconstruction in R to infer body size evolution throughout Ornithodira, discovering a distinct miniaturisation event near to the ornithodiran base.

This animal indicates the existence of a miniaturisation event occurring during the Triassic period, close to the evolution of dinosaurs and pterosaurs. The evolution of an extremely small body size enabled a trophic shift, opening up diversity in feeding strategies that included insectivory. The macroevolutionary implications run deeper, however. Small body sizes typically require different adaptations to those seen in larger animals, such as retention of body temperature and offer additional locomotor advantages. This miniaturisation event may provide insight about the evolution of fuzzy body coverings and flight in both pterosaurs and dinosaurs.

With many recent discoveries increasing the specimen resolution at this key time in archosaur evolutionary history, the tiny *Kongonaphon* is sure to influence thinking in this field in a big way. In the future, this may drive new studies into the evolution of varied body size and associated adaptations in this clade.
